# HSV-2-Specific Human Female Reproductive Tract Tissue Resident Memory T Cells Recognize Diverse HSV Antigens

**DOI:** 10.3389/fimmu.2022.867962

**Published:** 2022-03-31

**Authors:** David M. Koelle, Lichun Dong, Lichen Jing, Kerry J. Laing, Jia Zhu, Lei Jin, Stacy Selke, Anna Wald, Dana Varon, Meei-Li Huang, Christine Johnston, Lawrence Corey, Christine M. Posavad

**Affiliations:** ^1^ Department of Medicine, University of Washington, Seattle, WA, United States; ^2^ Department of Laboratory Medicine and Pathology, University of Washington, Seattle, WA, United States; ^3^ Vaccine and Infectious Diseases Division, Fred Hutchinson Cancer Research Center, Seattle, WA, United States; ^4^ Department of Global Health, University of Washington, Seattle, WA, United States; ^5^ Department of Translational Research, Benaroya Research Institute, Seattle, WA, United States; ^6^ Department of Epidemiology, University of Washington, Seattle, WA, United States

**Keywords:** HSV-2, CD8, CD4, epitope, dendritic cell, female reproductive tract immunology

## Abstract

Antigen-specific T_RM_ persist and protect against skin or female reproductive tract (FRT) HSV infection. As the pathogenesis of HSV differs between humans and model organisms, we focus on humans with well-characterized recurrent genital HSV-2 infection. Human CD8+ T_RM_ persisting at sites of healed human HSV-2 lesions have an activated phenotype but it is unclear if T_RM_ can be cultivated *in vitro*. We recovered HSV-specific T_RM_ from genital skin and ectocervix biopsies, obtained after recovery from recurrent genital HSV-2, using *ex vivo* activation by viral antigen. Up to several percent of local T cells were HSV-reactive *ex vivo.* CD4 and CD8 T cell lines were up to 50% HSV-2-specific after sorting-based enrichment. CD8 T_RM_ displayed HLA-restricted reactivity to specific HSV-2 peptides with high functional avidities. Reactivity to defined peptides persisted locally over several month and was quite subject-specific. CD4 T_RM_ derived from biopsies, and from an extended set of cervical cytobrush specimens, also recognized diverse HSV-2 antigens and peptides. Overall we found that HSV-2-specific T_RM_ are abundant in the FRT between episodes of recurrent genital herpes and maintain competency for expansion. Mucosal sites are accessible for clinical monitoring during immune interventions such as therapeutic vaccination.

## Highlights

HSV-specific T cells persistently localize to the female reproductive tract in humans.

Female reproductive tract T cells recognize specific HSV peptides.

Female reproductive tract T cells readily expand *in vitro* with standard conditions.

## Introduction

The majority of human T cells reside in mucosal barrier tissues outside the vasculature, and have a motility and adhesion molecule profile limiting re-entry into the circulation (1). These cells, termed tissue-resident memory (T_RM_) lymphocytes, are heterogeneous and include conventional T cells with hypervariable CDR3 sequences. Some innate lymphoid cells with conserved TCR variable gene usage and CDR3 amino acid (AA) domains such as mucosal-associated invariant T (MAIT) and invariant natural killer T (iNKT) cells also have memory characteristics and are included in the broad category of tissue-resident lymphocytes (2). T_RM_ can recognize pathogens with high sensitivity and specificity. Emplacement of cells elicited by vaccination in barrier tissues is an aspirational goal for vaccines (3).

Herpes simplex virus (HSV) infection of mice provides a valuable T_RM_ model. After recovery from primary cutaneous or female reproductive tract (FRT) infection, the natural attenuation of HSV in mice prevents ganglionic reactivation and recurrent epithelial replication. Skin and FRT T_RM_ persist without re-exposure to antigen (4). Skin CD8+ T_RM_ specific for an immunodominant MHC-I-restricted peptide localize to the epidermis in the region of primary infection, and mediate local protection from exogenous re-challenge. HSV-specific CD4 T cells occur mostly in the dermis after recovery from infection and are not as restricted to the site of primary infection (5, 6). In the FRT, after priming by non-lethal primary infection, protection against challenge is mediated by local CD4 T cells (7). HSV-specific CD8 T cells can also protect mice if pulled from the circulation to establish FRT T_RM_ (3).

Human HSV T_RM_ studies are important because HSV pathogenesis in the natural host differs from that observed in experimental animals. Acute lethality upon primary infection is only observed in persons with profound global immunodeficiency, or variations in genes most closely associated with innate immunity (8). In naïve mice, in contrast, FRT inoculation with wild-type HSV-2 is almost always fatal (9). Infected persons have periodic epithelial recurrences, with HSV-2 DNA detectable in the genital tract a median of 17% of days, in many cases without lesions or symptoms (10). These recurrences are initiated by viral reactivation in sensory ganglia, which are also a relevant local battlefield involving antigen-specific CD4 and CD8 T cells in both mice and humans (11, 12). In mice, spontaneous or even immune deficiency-triggered recurrences are very unusual and seldom progress to lytic viral replication in the periphery. Overall, periodic re-exposure to HSV antigen in humans could condition skin and FRT T_RM_ in ways that differ from the single priming episode in murine models.

We previously found that HSV-specific CD4 and CD8 T cells can be detected in cervical specimens, even during receipt of suppressive antiviral medication (13, 14). Genital skin biopsies also revealed preferential deposition of CD8α+ cells at the DEJ of healed lesion skin compared to normal skin. Laser capture microdissection (LCM) and immunohistology showed that these cells had low chemokine receptor and mobility-associated mRNA expression and expressed cytolytic and antiviral cytokine transcripts (15). Some DEJ CD8α+ cells stained *in situ* with fluorescent multimer reagents comprised of HSV peptides and subject-matched HLA class I, identifying them as antigen-specific CD8+ T_RM_. *TRCB* CDR3 sequences from LCM-captured DEJ CD8α+ cells from serial biopsies over several years revealed persistent oligoclonal expansions consistent with the localization of clonally expanded T_RM_ (15). In the present report, we build on these previous findings by more precisely measuring the frequency of HSV-2-reactive CD4 and CD8 T cells directly *ex vivo* in FRT specimens obtained at times when HSV is absent, and by determining their antigenic breadth and fine specificity.

Attempts to boost local T cells by therapeutic vaccination of humans have had measurable clinical success as quantified by reduction in genital lesions and HSV shedding (16, 17). However, it is not known if the bivalent approach used, containing two of the more than 75 proteins in the HSV-2 genome, contains the optimal viral antigens. Strategies to determine T cell specificity from minute biological specimens typically rely on *in vitro* expansion prior to interrogation of the relevant proteome (18). The complex HSV-2 genome and HLA diversity, present challenges to both *in situ* or direct *ex vivo* approaches as well as interrogation of T cell lines after expansion. Some data indicate that T_RM_ can proliferate *in vivo* (19), but the capacity of these cells to expand *in vitro* for detailed studies is poorly understood, as T_RM_ have specific metabolic and cytokine requirements (20). For example, lipids are important for human skin T_RM_ energy metabolism (21). To explore the hypothesis that some HSV-specific T_RM_ can expand *in vitro*, we started with a dendritic cell (DC)-based activation-induced marker (AIM) step to both measure and enrich virus-reactive skin T cells, based on a method used for blood (18). T_RM_ were then interrogated for fine specificity with HSV genome-wide tools tailored to CD8 and CD4 T cell phenotypes.

## Materials and Methods

### Subjects and Specimens

Women seropositive for HSV-2 with a history of genital herpes were enrolled at the University of Washington Virology Research Clinic (VRC). Participants reported the number of annual genital herpes recurrences and the time of their most recent recurrence. Most participants collected anogenital swabs daily for at least 30 days for HSV detection by PCR (10) to measure HSV shedding rate. Serum antibodies to HSV-1 and HSV-2 were detected by immunoblot (22). Peripheral blood mononuclear cells (PBMC) were cryopreserved (23). Skin biopsies (3 mm) at sites of previous genital HSV-2 recurrence were performed as described (24). A speculum was used to visualize the cervix. Biopsies of the ectocervix were performed using a Baby Tischler forceps. Up to 3 biopsies were obtained at a single visit. Biopsies were digested with pre-warmed Collagenase Digestion Medium with DNAse I (Sigma Aldrich) at 37°C for 30 minutes. HLA typing (**Supplementary Table 1**) was performed at Bloodworks Northwest or Scisco (both Seattle, WA) using PCR or sequencing-based methods (25).

A cohort of six additional HSV-2 infected, immunocompetent women was recruited to provide cervical cytobrush samples and PBMC as previously described (14, 26). Specimens were negative for HSV DNA on the day of collection. Characteristics of participants donating cytobrush specimens are reported in **Supplementary Table 2**.

### AIM Enrichment of HSV-2-Reactive Biopsy T Cell Lines

*In vitro* incubations were performed in humidified 5% CO_2_ at 37°C. Autologous monocyte-origin dendritic cells (moDC) were initiated from PBMC 6 days pre-biopsy as described (18). The protocol uses the ability of moDC to cross-present complex antigen to CD8 T cells and to simultaneously process and present complex antigen to CD4 T cells. HeLa cells (ATCC, Manassas, VA CCL-2) were infected with HSV-2 333 (Genbank KP192856.1) deleted of gene *UL41* (J. Blaho, City University of New York) at MOI 2.5 for 18 hours. The protein product of *UL41* has immune evasion effects on dendritic cells (27, 28). Infected or mock HeLa recovered with PBS-2 mM EDTA were treated as described with ultraviolet-C light (18). DC were seeded at 2 X 10^5^ cells/well in 48-well plates, to which an equal number of HeLa cells were added in a total of 300 μl T cell medium (TCM) (29). After five hours for processing of HeLa cell-based antigen by moDC, biopsy digest cells were added for a final volume of 600 μl. In a separate well, biopsy-derived cells were added to moDC that were not pre-loaded with HeLa-cell based antigen, with 1.6 μg/ml PHA (Remel, Lenexa, KS) used as a positive control stimulus. After 18-20 hours, cells were recovered and stained with 7-AAD viability marker, anti-CD3-energy coupled dye (ECD) (Beckman Coulter IM2705U), anti-CD4-allophycocyanin cyanine-7 (allophycocyanin-Cy7) (Biolegend 344616), and anti-CD8-FITC (Life Technology MHCD08014). Anti-CD137-allophycocyanin (BD 550890) was used to detect T cell activation (30). Live, CD3(+)/CD4(+)/CD8(–) or CD3(+)/CD4(–)/CD8(+) cells that were CD137(+) were bulk-sorted (FACSAria III, BD).

CD137^high^ cells were initially polyclonally expanded with PHA, feeder cells, and human natural IL-2 as described (31). Feeder cells assist T cell expansion when a non-specific mitogen is used to stimulate cell division (32). After ~ 14 days, cells were cryopreserved, or if reagents were ready (below), functionally tested and re-expanded. Polyclonal re-expansion used anti-CD3, feeder cells, and rIL-2 (33). As an alternative FRT specimen, cervical cytobrush-derived T cells (13) were recovered as previously described (14) and bulk-expanded using anti-CD3, cytokines and feeder cells (29) with neither AIM enrichment *ex vivo* nor *in vitro* exposure to HSV antigen. The rationale for non-specific T cell expansion was provision of enough FRT T cells to conduct proteome-wide determination of specificity.

### T Cell Functional Assays

For CD8 T cell lines (TCL), intracellular cytokine staining (ICS) assays were used for initial measurement of specific recognition of whole HSV-2. TCL and autologous EBV-LCL were plated at 5 X 10^5^ each in 96-well U bottom plates. EBV-LCL were uninfected or pre-infected with HSV-2 strain 186 (Genbank JX112656.1) at MOI 2.5 for 24 hours. Co-stimulatory anti-CD28 and anti-CD49d mAb were added immediately, and Brefeldin A was added at 2 hours (34). While it was possible that some EBV-specific T cells might be present in the cultures, we included non-infected EBV-LCL as a negative control to check for this. The re-expansion of TCL described above was not done after TCL exposed to HSV-2-infected EBV-LCL, but was done on non-manipulated cells after their initial CD137-based selection and expansion, such that amplification of EBV-specific T cells was avoided. After 18 hours, were cells stained with Fixable Live/Dead Near IR (ThermoFisher), treated sequentially with FACS Lyse and Permeabilization 2 (BD), stained with pooled antibodies to CD3-ECD, CD4-PE (Biolegend), CD8-FITC, IL-2-allophycocyanin (BD), and IFN-γ-PE-Cy7 (BD), washed and analyzed (Canto RUO, BD). PHA was used as positive control. Percentages of live, CD3(+)/CD8(+)/CD4(-) T cells expressing IFN-γ, IL-2, or both was recorded. Functional measurement of biopsy-derived CD4 TCL reactivity with whole HSV-2 used similar ICS conditions, tailored for CD4 T cells using autologous PBMC as APC and inactivated HSV-2 as antigen. Monocytes in PBMC can internalize and process killed HSV-2 for presentation to CD4 T cells (35). PBMC used as APC were pre-labeled with Cell Tracker Violet™ (CTV, ThermoFisher) to allow de-gating during ICS. PBMC and CD4 TCL were co-incubated at ~ 5 X 10^5^ each in 200 μl TCM. Antigens included UV-treated HSV-2 186-infected or mock UV-treated Vero cell lysates (1:100), and PHA positive control. Analysis gated on live CTV(-)/CD3(+)/CD4(+)/CD8(-) T cells for expression of IFN-γ, IL-2, or both.

### HSV-2 Specificity

Published methods (18) previously used to determine antigenic HSV-1 open reading frame(s) (ORFs) and peptide epitopes were modified for HSV-2. Each HSV-2 ORF from HSV-2 186 (Genbank JX112656.1) was PCR-cloned into a Gateway™ donor vector (Invitrogen) and shuttled using Gateway Clonase™ reactions into pDEST203 and expressed by *in vitro* transcription/translation (IVTT) for CD4 T cell studies. HSV-2 PCR primers, clone identities, and clone shuttling strategies are published (36). For CD8 T cell work, the same HSV-2 ORFs in donor vectors were shuttled into pDEST103 (18). Long HSV-2 ORFs cloned as fragments (36) included *UL19*, encoding major capsid protein VP5, and *RS1*, encoding infected cell protein 4 (ICP4), which were expressed as two and three overlapping fragments, respectively (36).

To screen biopsy-derived TCL for protein-level CD4 T cell responses, responder cells and autologous irradiated PBMC as APC (10^5^/well each), and IVTT proteins at 1:2000 were incubated in duplicate in 200 μl TCM for 3 days. Negative controls were *P. falciparum, F. tularensis*, or vaccinia virus (VV) proteins (37, 38), empty pDEST203 vector IVTT products, IVTT reaction mix without plasmid, UV-mock virus, and media. Positive controls were PHA and UV-treated HSV-2-infected Vero cell lysate. Proliferation was assessed by ^3^H thymidine incorporation (24). The statistical cutoff used to establish positive reactivity has been described (39). To screen bulk cervical cytobrush-derived T cells for CD4 T cell responses, duplicate lymphoproliferation assays in 96-well U bottom plates contained 10^5^ each cervical T cell responders, autologous gamma-irradiated PBMC, and antigens in 200 μl TCM. Each HSV-2 gene or fragment expressed by IVTT was added at 1:1000 together, with negative controls and PHA-P (1.6 μg/ml) as positive control. Proliferation assays were performed as for biopsy-derived TCL.

To screen biopsy-derived TCL for CD8 T cell responses, participant-specific HLA A and B cDNA alleles (**Supplementary Table 1**) were PCR-amplified and then cloned into pCDNA3.1 as described (33). HLA sequences were confirmed as correct using BLAST comparison to sequences in the IMGT database (40). Only the HLA alleles matching individual participants were used in functional screens. Artificial APC (aAPC) were created by simultaneously transfecting Cos-7 cells in 96-well flat-bottom plates with a single HLA cDNA and HSV-2 ORFs cloned into pDEST 103 (18). Each ORF-HLA combination was tested in duplicate. Polyclonal CD8 TCL were tested at 5 X 10^4^ cells/well two days after HLA/HSV-2 gene co-transfection. Supernatants were tested for IFN-γ secretion by ELISA at 48 hours (33).

### Peptide-Level Specificity

Once CD4 T cell reactivity was detected, overlapping peptides (OLP) (Genscript, or New England Peptide, Gardner, MA, 70% purity) covering the relevant ORF were tested. Some OLP are published (41). Peptides were dissolved and stored at 10-20 mg/ml in DMSO. Peptides were 13 to 15 AA overlapping by 9-11 AA (41), pooled at up to 20 peptides/pool as matrix pools (42), and tested at final concentrations of 1 μg/ml each. Assays used 5-10 X 10^4^ TCL and 5-10 X 10^4^ autologous irradiated (8000 rad) EBV-LCL/well as APC (42). Single peptides at the intersection(s) of reactive row and column pools that gave positive responses were tested with the same responder cells and APC. T cell activation was determined using ^3^H thymidine proliferation, secreted IFN-γ, or IFN-γ/IL-2 ICS as described (18).

For CD8 T cell fine specificity, OLP were also tested as matrix pools followed by decoding to single peptides. Autologous EBV-LCL and bulk CD8 T cells (10^5^/well each) were admixed with peptide pools or single peptides (1 μg/ml each peptide) in 96-well U bottom plates in 200 μl TCM overnight and supernatants assayed for IFN-γ. As an alternative to OLP, predictive algorithms for the relevant HSV-2 ORF and HLA allele (43) were consulted and peptides with high predicted HLA binding affinity were synthesized (Genscript). Single peptides from reactive pools or algorithmic prediction were tested using IFN-γ ELISA or ICS (18).

## Results

Six HSV-2 seropositive women with a median age or 43 (range, 38 to 62) with a history of recurrent genital herpes were enrolled in a prospective biopsy protocol (**Table 1**). Participants had clinical histories of genital HSV for a median of 19 years prior to specimen collection (range, 1 to 33 years). The median annual number of self-reported recurrences of genital herpes was 2 (range, 1-6), typical for immune competent persons (10). Each participant collected daily genital swab specimens for shedding studies as described (10) and each swab was used for HSV-specific DNA PCR. The median viral DNA detection rate of 22.3% (range, 4.4% to 55.7%) was similar to the median rate of 17% of days determined from large natural history studies (44). Genital swabs at six of the eight times of specimen collection were negative for HSV DNA by PCR, with data not available at two times of biopsy specimen collection.

**Table 1:** Characteristic of participants and specimens.

PtID	Biopsy date(s)	Age	HSV-1^1^	Duration of genital herpes at time of biopsy, years	genital herpes recurrences/ year^2^	genital HSV shedding rate^3^	cervical specimen	genital skin specimen, location	day(s) since most recent clinical herpes recurrence(s)^4^
9149	8/31/16	54	negative	31	2	8.3	yes	perineum	No information
13497	11/2/2016	62	negative	33	6	46.6	yes	perineum	28
14655	10/31/2016	38	negative	20	2	29.5	yes	left perineum	No information
14887	10/27/2016	43	positive	14	1	4.4	yes	right labia	5, 64
15018	6/27/2016	37	negative	1	2	57.7	yes	left perineum	8
15018	9/28/2016			1			yes	left perineum	10
15052	5/5/2016	44	positive	3	1	15.1	yes	not collected	No information
15052	6/9/2016			3			yes	left perineum	No information

^1^ Presence of serum anti-HSV-1 IgG. All participants are seropositive for HSV-2.

^2^ As reported by participants.

^3^ Measured by PCR detection of mixed anogenital swabs, reported as percent of days positive, in the 30 days following biopsies.

^4^ Days between healing of most recent genital lesion or lesions and biopsy.

### Healed Genital HSV-2 Skin and Ectocervix Contain Culture-Expandable HSV-2-Reactive T Cells

**Figure 1:**
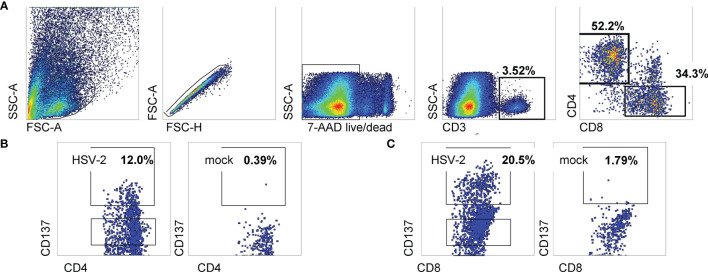
Sorting data for cervical T cells from subject 15018 initial cervix biopsy. **(A)** Gating scheme. **(B, C)** Gated CD8 and CD4 T cells stained for CD137 expression after exposure to HSV-2- or mock-loaded autologous moDC. Gates show cells selected for sorting as CD137^high^. Percentages are proportion of cells in dotplots within CD137^high^ gates.

Cells from enzymatically digested biopsies were co-cultured with autologous blood-derived, monocyte-origin DC (moDC) that were loaded with whole HSV-2 antigen in the form of infected, UV-treated HeLa cells. The protocol allows antigen uptake, processing and cross-presentation to CD8 T cells, as shown in our work on HSV-1 and vaccinia (18). The HSV-2 strain used to antigen-load moDC lacks *vhs*, a protein that inhibits DC function (28). Data from the June biopsy from a representative sample from participant 15018 show a minority (3.52%) of cervical cells were CD3(+) T cells (**Figure 1A**). A discrete population of CD4 T cells expressed the activation marker CD137 (also called 4-1BB and TNFRSF9) after 18 hours exposure to HSV-2-loaded DC, with much lower responses to mock-loaded DC (**Figure 1B**). Similarly, specific activation of CD8 T cells was noted (**Figure 1C**). Similar trends were noted for the majority of FRT specimens (**Table 2**). Activated cells were expanded with non-specific mitogens. Overall, 29 biopsy-derived T cell lines derived were studied, including paired CD4 and CD8 T cell lines from 7 genital skin biopsies, and 8 CD8 and 7 CD4 cervix-derived T cell lines (**Table 2**). Up to 12% of CD4 T cells and 20.5% of CD8 T cells were HSV-2-reactive (subject 15018, initial cervical biopsy, **Table 2**). Even if analytical flow cytometry did not show net HSV-2-specific CD137 expression response for either or both T cell subsets, CD137^high^ CD4 or CD8 T cells were isolated for downstream studies.

**Table 2:** Activation of FRT biopsy-derived T cells after exposure to HSV-2-loaded autologous moDC.

PtID	biopsy date(s)	Biopsy site			
		Cervix		Skin	
		CD3+ T cell phenotype		CD3+ T cell phenotype	
		CD4+CD8-	CD8+CD4-	CD4+CD8-	CD8+CD4-
9149	8/31/16	0.9^1^	4.7	0.4	5.6
13497	11/2/2016	0.8	0	0.9	0
14655	10/31/2016	5.1	1.7	0	0
14887	10/27/2016	1.3	1	0.4	1.4
15018	6/27/2016	12	20.5	0	0
15018	9/28/2016	3.7	3.5	0	0
15052	5/5/2016	ND^2^	0.3	ND	ND
15052	6/9/2016	0	2.4	0.8	2.5
mean		3.40	4.26	0.36	1.36
standard deviation		4.20	6.75	0.38	2.1

^1^Values are net percentage of biopsy-derived live T cells expressing CD137 for HSV-2-loaded DC minus mock virus-loaded DC. Net values of less than zero are reported as zero.

^2^ND indicates not done.

**Figure 2:**
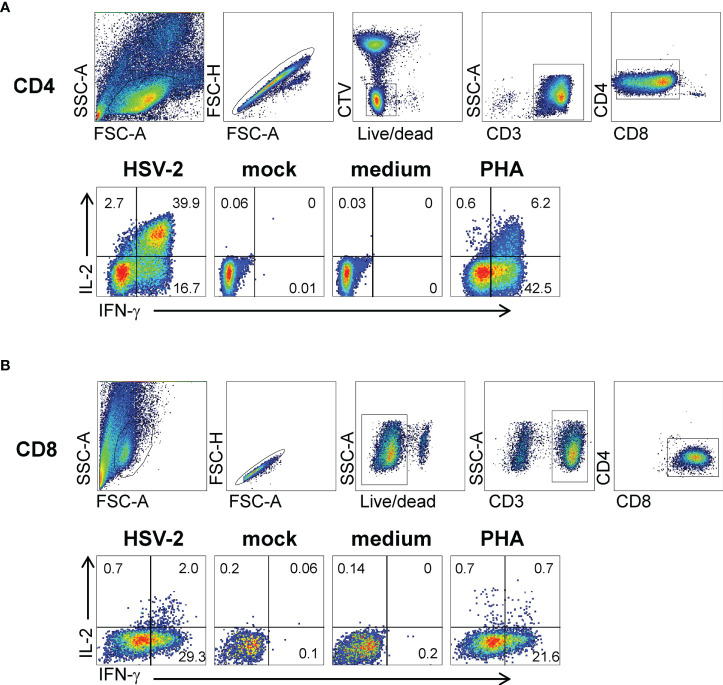
Responses of bulk-expanded, AIM-selected TCL from subject 15018 June cervix biopsy. **(A)** CD4 T cells. Top row shows gating scheme. Autologous PBMC used as APC were dump-gated by prior labeling with CTV. Lower row has gated CD4 T cells analyzed by ICS for accumulation of IFN-γ and IL-2 with the labeled stimuli. Specific responses are present to autologous PBMC treated with UV-HSV-2 antigen for CD4 responder cells. **(B)** CD8 T cells. Upper row has gating scheme. EBV-LCL used as APC are CD3-negative and did not require dump gating. Lower row shows responses to autologous EBV-LCL infected with HSV-2 or mock virus, and control stimuli. Numbers are percentages of gated cells.

Activated CD137^high^ CD4 or CD8 T cells sorted from HSV-2-stimulated conditions were polyclonally expanded and the reactivity of the resultant bulk TCL measured using appropriate APC. Experiments to document enrichment of HSV-2-reactive CD4 T cells within the CD137^high^-origin CD4 TCL used autologous PBMC as APC, whole viral antigen, and IFN-γ/IL-2 ICS as readout. Marked enrichment was noted (representative data in **Figure 2A**). Both IFN-γ and IL-2 were strongly positive. Similar enrichment measurement was performed for biopsy-derived CD8 TCL. HSV-2-infected EBV-transformed B lymphocytes (EBV-LCL) were used as APC to allow for endogenous antigen processing and presentation. While B lymphocytes are not targets of HSV infection *in vivo* (45), EBV-LCL are permissive for lytic HSV replication *in vitro* (46) and are recognized by CD8 HSV-2-specific T cells (31). Specific recognition of whole HSV-2 resulted in high frequencies of IFN-γ-expressing CD8 T cells. IL-2 also detectable (**Figure 2B**) but at levels lower than for CD4 T cells.

### Biopsy-Derived T_RM_ Recognize Discrete HSV-2 Antigens and Epitopes

**Figure 3:**
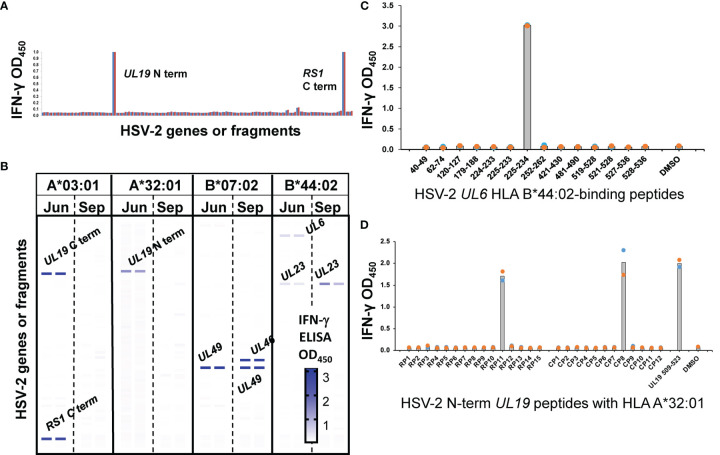
CD8 T cell recognition of HSV-2 antigens by AIM-enriched cervical biopsy-derived T_RM_ from subject 15018. **(A)** Representative duplicate data for June biopsy. Rightmost bars are negative controls (empty vector plus HLA, HLA alone). aAPC express HLA A*03:01, a single subject-specific HLA class I allele and individual HSV-2 genes or fragments **(B)** Heat map of reactivity with duplicate assays for subject HLA **(A, B)** Responder cells were derived from June (Jun) and September (Sep) biopsies. Reactive HSV-2 genes are labeled. Negative control stimuli in bottom two rows. Screens for each subjects-specific HLA A and B allele (A*03:01, A*32:01, B*07:02, and B*44:02) are shown separately with the intensity of IFN-γ expression indicated per the graphic scale. **(C)** CD8 T cell epitope discovery using predicted HLA -binding peptides. TCL from June biopsy assayed with 1 μg/ml indicated *UL6* peptides and autologous LCL as APC. **(D)** OLP pathway for epitope discovery. TCL from same biopsy stimulated with matrix row (RP) and column (CP) pools of 10 to 20 15 AA-long peptides/pool spanning the reactive UL19 AA 1-703 fragment at 1 μg/ml each peptide. At right, results for 1 μg/ml peptide at the intersection of RP11 and CP8 pools. Colored dots are duplicate raw data and gray bar is mean.

Virus-specific cytokine responses to whole HSV-2 by polyclonal FRT biopsy-derived TCL suggested the presence of reactivity to detect discrete viral proteins and internal peptides, and restriction by autologous HLA alleles. To study this, CD8- and CD4-specific methods from HSV-1 research (11, 18) were adapted for proteome-wide screens of HSV-2. For CD8 T cells, panels of artificial APC (aAPC) were created using Cos-7 cells, a well-established procedure in which the non-human primate Cos-7 provide a compatible β2-microglobulin partner for HLA class I (47). The aAPC were co-transfected with HLA and a panel of plasmids encoding each HSV-2 gene. A representative CD8 TCL from participant 15018 for one exemplar HLA allele, HLA A*03:01, show recognition of the HSV-2 *RS1*-encoded protein ICP4, as well as the N-terminal fragment of the *UL19*-encoded major capsid protein VP5 (**Figure 3A**) using an IFN-γ readout, with negligible background responses. The same TCL showed HLA A*32:01-restricted recognition of the C-terminal portion of VP5, HLA B*07:02-restricted recognition of VP22 (encoded by *UL49*), and HLA B*44:02-restricted recognition of the *UL6*-encoded capsid portal protein and the *UL23*-encoded thymidine kinase. Overall, results from screens of each subject-specific HLA A and B allele show that the TCL from this cervical biopsy recognized 6 separate HSV-2 proteins (**Figure 3B**). To determine if T_RM_ persisted over time, we obtained cervical biopsy from this participant 3 months later (**Figure 3B**). *UL49* and *UL23-*specifc responses with the same HLA restriction were again noted. A new specificity (HLA B*07:02-restricted reactivity to *UL46*-encoded tegument protein VP11/12) was detected at the later time point.

Following antigen identification, internal discrete CD8 T cell epitopes were documented for selected TCL and HLA cDNA/HSV-2 ORF combinations. In some cases, assays focused on peptides in reactive HSV-2 ORFs predicted to bind relevant HLA alleles (representative data, **Figure 3C**). In other cases, ORF-covering OLP sets were tested, typically as matrix pools followed by single reactive peptides (representative data, **Figure 3D**). Additional examples of CD8 T_RM_ HSV-2 peptide epitope discovery (**Supplementary Figure 1**) show high signal-to-noise ratios and discrete recognition of single peptides. We note that for CD8 and CD4 workflows (below), the discovery of discrete epitopes within “hit” HSV-2 ORFs serves functionally as validation of T cell reactivity with the ORF in question, contributing to rigor and reproducibility. Further confirmation of accurate identification of antigenic specificity is afforded by peptide dose-response analyses (also below) in which reactivity with peptides within HSV-2 ORFS is repeatedly observed.

**Figure 4:**
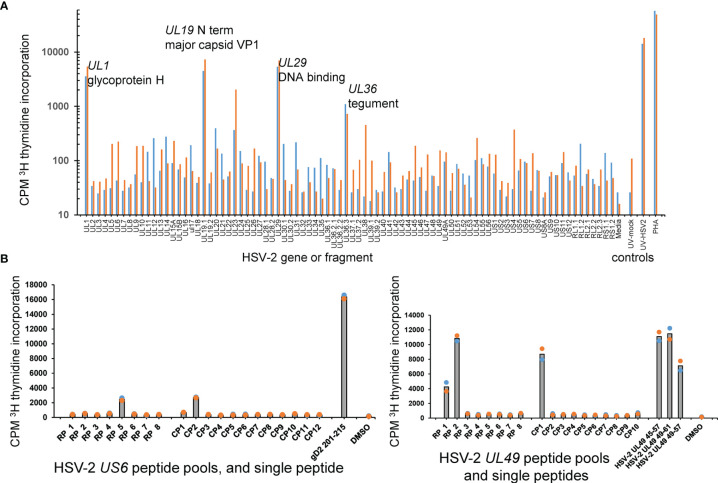
HSV-2 FRT CD4 T cell antigen and epitope discovery using culture-expanded T_RM_. **(A)** Screen of skin biopsy-derived TCL from subject 9149 against HSV-2 proteome in duplicate proliferation assay. Gene and short protein names of antigenic proteins are shown; negative control mock virus and positive control whole UV-killed HSV-2 and PHA at right. **(B)** Peptide epitope workup for cervix-derived TCL from subject 9149. OLP from HSV-2 gD (gene *US6*, 15AA long, left) or HSV-2 VP22 (gene *UL49*, 13 AA long, right) were arrayed in row (RP) and column (CP) pools with 1 μg/ml each peptide. Peptides from the intersection (*UL6*) or intersections (*UL49*) of reactive pools were re-assayed at 1 μg/ml at right of each diagram. APC were autologous irradiated PBMC. Colored dots are duplicate raw data and gray bar is mean.

The AIM-enriched biopsy CD4 TCL were similarly tested using a proteome-covering panel of recombinant HSV-2 proteins (36). Given the requirement for antigen uptake, processing and presentation to CD4 T cells, autologous PBMC were used as APC. A cell proliferation assay optimized in our previous viral proteome-wide screens (18, 48, 49) was used in place of IFN-γ secretion, albeit we have also used IFN-γ secretion to probe T cell responses to protein panels from microbial genomes (38). CD4 T_RM_ reactivity with individual HSV-2 proteins was frequently detected. Representative data from the skin biopsy of participant 9149 (**Figure 4A**) showed reactivity for at least four HSV-2 proteins: *UL1*-encoded glycoprotein L, the *UL19* major capsid protein, the *UL29* DNA binding protein, and the *UL36* large tegument protein.

We separately measured HSV-2 antigen-level specificity of a cervical cytobrush-derived bulk TCL. With these specimens, HSV-2-reactive cells were not enriched *ex vivo* by antigen re-exposure or selection *in vitro*. Two participants (9149, 15018, **Table 1**) overlapped with the biopsy cohort and an additional 6 persons were also studied (**Supplementary Table 2**). Discrete responses to individual HSV-2 proteins were frequently detected (representative data, **Supplementary Figure 2A**). These responses are presumed to be due to CD4 T cells as presentation of IVTT-expressed proteins by APC in unmanipulated PBMC to CD8 T cells is unlikely. Overall, at least one HSV-2 protein was recognized in all 14 cytobrush samples from each participant (**Supplementary Figure 2B**). Across the specimen set, 34 of the 71 HSV-2 proteins were antigenic in one or more samples. Within-participant consistency was noted for reactivity to some HSV-2 proteins; for example, participant 13931 recognized HSV-2 proteins UL23, UL39, and UL46 in each of 3 cytobrush specimens collected over 263 days. These results extend previous HSV-2 genomic library and peptidome surveys (26, 35) and indicate that both deeper biopsies of cervical tissue and superficial cells accessible to cytobrush scraping are rich in HSV-2-specific CD4 T cells.

Once reactive HSV-2 ORFs were identified, specific CD4 T cell epitopes were determined using OLP approaches as for CD8 T cells, above. Representative data for the cervix-derived TCL from participant 9149 (**Figure 4B**, left) shows an example from *US6* (encodes glycoprotein D) in which single matrix pools were reactive. The peptide nominated by the pool approach, gD AA 201-215, was confirmed as reactive in a follow-up assay. Other matrix results were more complex. In an example for the same TCL and HSV-2 *UL49*, column pool 1 and both row pools 1 and 2 were reactive. The 13 AA peptides *UL49* 45-57 and *UL49* 49-61, at the respective intersections of these pools, were each antigenic. As expected, the 9 AA peptide *UL49* AA 49-57, at the overlap of the antigenic 13 AA peptides, was also recognized by the TCL (**Figure 4B**, right). For CD4 TCL from subject 9149, IFN-γ ICS epitope mapping using peptide pools agreed quite well with pool-level and follow-up single peptide proliferation assays (**Supplementary Figure 3**). IFN-γ ICS was also used for the same participant to confirm fine peptide specificity after initial pool-level mapping using proliferation assays (**Supplementary Figure 4**).

### CD8 T_RM_ Functional Avidity

**Figure 5:**
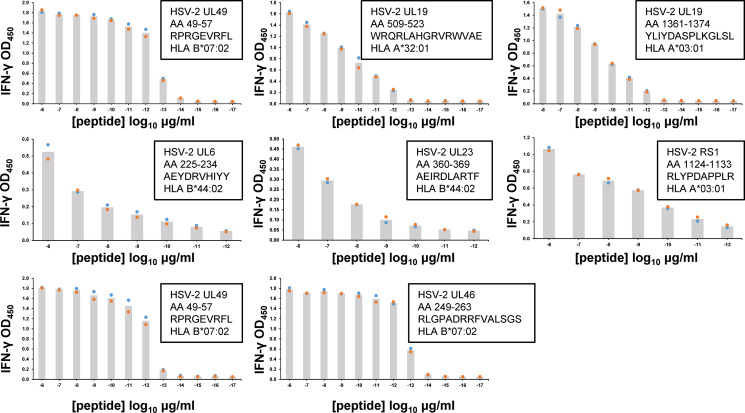
Functional avidity of human FRT-derived HSV-2-specific polyclonal CD8 T_RM_ from subject 15018 cervical biopsies from June (top rows) or September (lower row) samples. TCL were incubated with autologous EBV-LCL and dilutions of peptide. Insets list HSV-2 gene, AA numbers and sequence of peptide tested, and HLA restriction of epitope. T cell activation was measured by supernatant IFN-γ ELISA. Colored dots are duplicate raw data and gray bar is mean. Note varied X and Y axes. Some peptides were tested over an expanded concentration range. DSMO negative control <0.06 OD_450_ for all assays.

Following peptide epitope identification, the functional avidity of culture-expanded, polyclonal CD8 T_RM_ was evaluated using peptide titration. We concentrated on the serial cervical biopsies from subject 15018, studying T cells specific for every HSV-2 antigen/HLA combination detected as antigenic in screens of HLA/HSV-2 gene co-transfected aAPC (**Figure 3**). CD8 T_RM_ could show very high functional avidity (**Figure 5**). For example, UL49 AA 49-57-specific CD8 T_RM_ from cervical biopsies separated by 3 months had estimated EC_50_ values below 1 picogram/ml peptide. Response potency was typically higher than those observed for HSV-2 CD8 T cell clones from active HSV-2 lesions (33), derived from PBMC by tetramer sorting (50) or obtained from blood by leveraging the high, specific cutaneous leukocyte antigen (CLA) expression programmed into circulating memory HSV-2-specific CD8 T cells (51, 52). Remarkably, the same 9 amino acid peptide, UL49 AA 49-57, was potently recognized by both CD8 (**Figure 5**) and CD4 T cells (**Figure 4B**), albeit from separate participants.

## Discussion

This report focuses on the human FRT as an interface between HSV and the acquired immune response. For the cervix, we used a combination of biopsies and cytobrush samples to study both deeper and more superficial local T cell responses to this pathogen. In the context of intermittent HSV-2 antigen exposure in the natural host, we find that HSV-2-specific CD8 and CD4 T cells are abundant, retain the ability to recognize antigen by up-regulating activation markers *ex vivo*, and briskly proliferate *in vitro*. A broad spectrum of viral antigens and epitopes are recognized, often with high functional avidity.

The work in this report indicates that T_RM_ can recognize a relatively wide array of HSV-2 antigens. CD8 T cell antigens recognized by the skin and FRT-derived T cells in this report ranged from transcription factors (ICP4 encoded by *RS1*) expressed immediately after lytic replication starts, to abundant structural proteins such as the major capsid protein VP1 encoded by *UL19*, expressed late in the viral life cycle. The spectrum of CD4 T cell antigens was also quite wide, especially when the cervical cytobrush-derived T cells are included. We have previously detected HSV-2-specific CD8 T cells in cytobrush specimens (53) and proteome-wide tools could be applied to extend specificity determination to these samples. Within-subject diversity is hard to estimate given the limited anatomic samples we can access, but it is remarkable that up to six discrete CD8 T cell epitopes could be detected within a cervical biopsy as for subject 15018, and up to 14 HSV-2 antigens were recognized by CD4 T cells from a cervical cytobrush from subject 13931. Previously, we resorted to T cell cloning from biopsies or cytobrush samples and typically used traditional genomic HSV-2 DNA molecular libraries to discovery the fine specificity of tissue-derived HSV-specific T cells (33, 35, 54). Throughput is greatly enhanced using polyclonal TCL and virtual libraries of HSV ORFs, at the possible expense of missing rare responses. Previous measures of blood or tissue T cell epitope diversity for HSV-1 and HSV-2 indicated recognition of 20 or fewer epitopes/specimen (18, 37, 55, 56), fairly close to our current data.

Our co-transfection workflow determines HLA restriction in parallel to antigenic specificity for CD8 T cells. For CD4 T cells, established pathways for determining restricting HLA loci and alleles (57) are available. For HSV-2 UL49 AA 49-57, a known HLA B*07:02 restricted CD8 T cell epitopes we also noted recognition by CD4 T cells for subject 9149 (**Figure 4**). Submission of the HSV-2 sequence to algorithms (43) for the relevant subject’s HLA class I and II alleles does not reveal any internal peptide sequence/allele combinations with high predicted binding affinity.

Related to specificity and phenotype, virus-specific CD4 and CD8 T cells may function optimally in close proximity to one another. For example, we observed that culture-expanded CD8 T_RM_ expressed minimal IL-2 while CD4 T cells expressed this cytokine in abundance (**Figure 2B**). Our work disclosed coordinated local CD4 and CD8 virus-specific T cell responses in many specimens. Other properties, such as the affinity of T_RM_ TCRs for peptide-HLA, could also be vital, with the reasonable hypothesis that high T_RM_ affinity would lead to earlier detection of pathogen and thus better control. Affinity requires biochemical assays to measure, but can be approximated by functional avidity, as in this report. We observed quite high functional avidity for polyclonal TCL specific for peptides in the tegument proteins encoded by *UL46* and *UL49* (**Figure 5**), with strong recognition of 1 pg/ml viral peptide. It is likely that the epitope-specific response is heterogeneous at the TCR clonotype level, as we observed in studies of T cells specific for a tumor virus (58), and that functional affinities vary with TCR sequence. In a murine HSV model with localization of virus-specific CD8 T cells to latently infected ganglia, ganglionic T_RM_ had higher functional avidity than T cells specific for the same HSV epitope found in the lung, a non-infected site (59). In a murine model of cleared vaccinia infection, functional avidity varied between intestinal CD8 T_RM_ specific between intraepithelial and lamina propria layers (60). Future work will be required to determine if there is a hierarchy of functional avidity between blood and tissue compartments. While we did not study enough subjects to suggest nominating a specific HSV-2 ORF as vaccine candidate, it should be noted that chimeric multi—partial ORF and multi-epitope vaccines are increasingly entering clinical trials for both infection and cancer, including tuberculosis and melanoma (61, 62) and COVID-19 (clinicaltrials.gov NCT04776317). HSV epitope knowledge from the present and prior genital tissue-based studies ((63) combined with blood and neuronal ganglia studies (11) could allow a similar customized antigen to design a T cell-directed HSV vaccine immunogen.

Our studies suggest that the lymphocytic infiltrate seen after HSV infection is comprised at least in part by antigen-specific cells. After recovery from attenuated HSV infection, memory lymphoid clusters containing HSV-reactive CD4 T cells occur submucosally in murine FRT (7) and skin (64). In humans, a leukocytic infiltrate occurs in the endocervix in response to HSV (65), and CD4 and CD8 T cells infiltrate the dermis during and after infection (66). Less is known about the ectocervix. HSV-2 antigen remains in the epithelium regardless of anatomic location in skin vs. mucosal surfaces, and does not penetrate the basement membrane in the immunocompetent host (66). Recently, a plasma cell infiltrate in acute and healed HSV-2 genital skin lesions and local HSV-specific IgG have been reported (67). While the fine specificity of the local B lymphocytes is not yet known, it is possible that local antigen-specific T cells cooperate with B lymphocytes for antibody production

These studies are motivated by the unmet need for preventative and therapeutic HSV vaccines. Therapeutic and preventative vaccine clinical trials have used HSV-2-based candidates ranging in complexity from peptides to whole virus (68). Partial success has been demonstrated with preventative vaccine candidates that trigger CD4 T cells and antibody in humans (69–72), and data that passive antibody transfer can protect mice in an FcR-dependent manner against genital HSV-2 challenge (73, 74) has led to renewed interest in antibody-based vaccine prevention. The success of recombinant zoster vaccine (RZV) for recurrent infection by varicella zoster virus (VZV), a pathogen that shares tropism, sequence, and T cell epitopes with HSV (34), has re-invigorated therapeutic HSV vaccine efforts as well. In this regard, a bivalent gD2/ICP4 HSV-2 protein-based candidate showed efficacy as measured by genital HSV shedding and lesion rates (16, 17). The mechanisms of action of RZV and gD2/ICP4 remain unknown but are presumed to involve antibodies and/or CD4 T cells (75–78). To date, prevalent, durable, and quantitatively significant boosts of CD8 T cells have not been demonstrated in human HSV vaccine immunotherapy trials. We and others (79) hypothesize that HSV-2-specific CD4 and CD8 T cells at the site of recurrent infection are important for containing virus, and that ultimately, boosting the levels or enhancing the phenotype of peripheral virus-specific T cells could lead to clinical and virologic reduction of recurrence infection.

Human studies, while challenging, are of interest because HSV pathogenesis varies greatly between species. Non-human primates range from hyper-susceptible (80) to mostly resistant (81). Outbred rats (82), guinea pigs (83), and rabbits (84) can recover and have recurrences, but immunologic tools are limited. Inbred mice differ for HSV susceptibility per genetic background (85) and require hormonal treatment for primary FRT infection (86). Surviving animals have a coordinated humoral, CD4 and CD8 T cell response. HSV-specific T_RM_ are readily observed in the skin, FRT, and sensory ganglia in mice. Sophisticated tools allow precise localization, quantitation, expression profiling, and live cell monitoring during immune surveillance (87–90). However, HSV does not recur to the level of peripheral culture-positive viral reactivation outside of profound epigenetic manipulations (91). Therefore, explant, re-infection, and parabiosis models are used to study T_RM_ functionality. In contrast, HSV reactivates in essentially all infected persons (10). For immune competent persons with antibodies to HSV-2 and a clinical history of genital herpes, the population studied in this report, viral DNA is detected in anogenital swabs by PCR on a median of 17-20% of days (10).

The properties associated with effective control of recurrent HSV infection by T_RM_ are unknown. Mathematical models indicate that T_RM_ density threshold may be critical to determining if the outcome of neuronal delivery of HSV from axon termini is a brief period of low level viral shedding or exponential growth of virus in a clinically evident recurrent lesion episode (92). Cytokine released by T_RM_ such as IFN-γ are thought to mediate paracrine effects to induce a net anti-viral state in the vicinity (93). Localization near sites of HSV replication may also be critical. Our human *in situ* work has stained occasional CD8 T cells at the dermo-epidermal junction (DEJ) in skin biopsies from sites of previous HSV recurrences with HSV-specific peptide-HLA reagents (15).

This study has several limitations. We used moDC as APC because of their ability to cross-present to CD8 T cells and to also process and present antigen to CD4 T cells. Discrete DC subsets prime CD8 and CD4 T cells in murine HSV models (94, 95) and human moDC can present HSV antigen (96), but further research is required to determine the importance of DC in the context of human recurrent HSV infection. We used a strain of HSV-2 deleted in *UL41* to re-stimulate HSV-2-specific T cells *ex vivo*, such that T cell responses to the protein product of *UL41* would be missed. *UL41* encodes an RNAse with complex influences on HSV replication, pathogenesis and immune evasion (97–100); biases in the spectrum of T cells recalled by presentation of *UL41*-deleted HeLa cells are therefore possible. The *UL41* deletant is based on HSV-2 strain 333. This has a small number of predicted amino acid changes from HSV-2 strain 186 used for whole virus readouts and as the source for HSV-2 proteins. Each subject was infected with yet a 3^rd^ strain of HSV-2 that could also vary in amino acid sequence, possibly contributing to strain-specific responses (101). Overall, however, coding differences between HSV-2 strains are relatively minor (102, 103).

In addition, we studied a limited number of subjects and supplemented biopsies with cervical cytobrush specimens, a site related to, but immunologic distinct from the ectocervix. Only women were studied in this research restricted to the FRT; similar studies of male-specific tissues could be envisaged but are limited by the male genital biopsies. It could be argued that the T cells studied in this report are not challenged to persist in the periphery in the long-term absence of antigen. The tools created in the current study should enable future work to fully define the phenotype of FRT and skin HSV-specific T cells in humans, and compare and contrast them to T cells left behind by other pathogens that are permanently cleared, such as the related virus VZV. With regards to the requirement for antigen for maintenance of local virus-specific cells, we have previously shown that HSV-2-specific CD4 cells are enriched in cervical cytobrush specimens (13) and genital skin biopsies (66) during treatment with the antiviral drug acyclovir, a treatment (104) that reduces antigen.

We did not phenotype biopsy- or cytobrush-derived cells for T_RM_ markers such as CD69 or CD103 *ex vivo*, and refrained from staining for these markers after T cell expansion due to uncertainty about the effect of *in vitro* culture on their expression. Several lines of evidence suggest the cells studied in this report are T_RM_. Specimens were obtained at times of negative HSV PCR, a surrogate for the absence of HSV antigen. In our previous studies comparing cervical biopsy-derived CD4 T cells with and without expression of the T_RM_ marker CD103, a higher proportion of CD103(+) than of CD103(-) cells showed IFN-γ responses to whole HSV-2 antigen. Both the CD103(+) and CD103(-) fractions had higher IFN-γ expression than did blood CD4 T cells (14). Expression of T_RM_ markers such as CD103 and CD69 is heterogeneous (105, 106). Recently, we reported that cervical biopsy-derived T cells obtained at times of negative HSV PCR express high levels of mRNAs encoding CD69 and the integrin subunits of CD103. Moreover, these cells have considerable *TRB* CDR3 overlap with blood HSV-2-specific CD8 T cells, implying that they are HSV-2-specific (107). Future studies could combine single cell phenotyping at the protein or mRNA levels with TCR sequencing and determination of virus specificity by expressing TCRs in reporter cells, a process we have piloted for blood-origin HSV-specific T cells (34). Theoretically, FRT-derived cells could be fractionated per T_RM_ marker expression after isolation from biopsies or cytobrush specimens and prior to antigen exposure, but this would likely be technically challenging given the recovery of low cell numbers from these specimens.

It is possible that the HeLa cells used to load HSV-2 antigen into moDC could re-stimulate HPV-reactive T cells. HeLa express HPV18 E6 and E7 (108). **Figure 1** is an example in which stimulation of PBMC with moDC-mock-HeLa led to non-zero, CD137-expressing CD4 and CD8 T cells, consistent with our previous publication (18). HPV-specific T cells are rare: *in vitro* elicitation of HPV E6 or E7-reactive T cells from PBMC uses repetitive cycles of re-stimulation with high concentrations of peptides (109), and *in vivo* vaccinations use many cycles of peptide-loaded DC (110). It would be possible to query our HSV-reactive T cell lines with HPV expression constructs (111) to detect HPV-specific reactivity in our system.

We did not directly compare the abundance of HSV-specific T cells in PBMC and cervical specimens from the same subjects. In a previous study comparing direct *ex vivo* PBMC CD137-based AIM with IFN-γ ICS, the abundance of CD8 T cells reactive with HSV-1-loaded autologous moDC was under 1% (18). In a separate study of 67 HSV-2-infected persons, including serial sampling, with *ex vivo* ICS to detect IFN-γ, IL-2, or TNF-α, the median net abundance of CD4 T cells expressing any one or more cytokine in response to whole killed HSV-2 was 0.56% (112). The observed high abundances of HSV-2-reactive T cells in some of our genital biopsy specimens is evidence for local quantitative enrichment, which could be reinforced by study of simultaneously collected blood and local specimens.

In conclusion, the human FRT is richly and persistently infiltrated with HSV-2-specific CD8 and CD4 T cells in immunocompetent persons with a clinical history of recurrent genital herpes. These virus-specific effectors are readily detectable at both the ectocervix and in genital skin. Responses are typically poly-specific and have high functional avidity. Ultimately, measurement of the functional importance of epithelial T_RM_ in the control of recurrent HSV infection awaits clinical trials of vaccines capable of emplacing and increasing T_RM_. The technologies developed for this study should enable safe and efficient T_RM_ assays based on clinically acceptable biopsies or cervical cytobrush specimens to be used in clinical trials of next-generation vaccines (113) being evaluated for elicitation of anatomically relevant T cell responses.

## Author’s Note

Presented in part at the 42nd International Herpesvirus Workshop, Ghent, Belgium, July 2017, abstract 1.05.

## Data Availability Statement

The original contributions presented in the study are included in the article/**Supplementary Material**. Further inquiries can be directed to the corresponding author.

## Ethics Statement

The studies involving human participants were reviewed and approved by University of Washington Institutional Review Board. The patients/participants provided their written informed consent to participate in this study.

## Author Contributions

DK, CP, and LC conceived the study. DK wrote the paper and created data visualizations. CJ, DV, and AW collected the specimens. LD, LCJ, LJ, JZ, CP, MH and KL optimized tissue preparations and performed experiments. SS managed data. All authors contributed to the article and approved the submitted version.

## Funding

NIH grants AI030731 (DMK, CJ, AW), R01 AI042528 (JZ and LC), R01 AI134878 (JZ and LC), P30 CA015704 (core facilities), R01 AI091701 (CP), R21 AI083418 (CP), NIH contract 75N93019C00063 (DK).

## Conflict of Interest

DK, CP, and LC are co-inventors of patents concerning HSV vaccines owned and managed by their institutions. DMK receives research funding from Sanofi Pasteur concerning testing samples from a clinical trial of an HSV vaccine.

The remaining authors declare that the research was conducted in the absence of any commercial or financial relationships that could be construed as a potential conflict of interest.

## Publisher’s Note

All claims expressed in this article are solely those of the authors and do not necessarily represent those of their affiliated organizations, or those of the publisher, the editors and the reviewers. Any product that may be evaluated in this article, or claim that may be made by its manufacturer, is not guaranteed or endorsed by the publisher.

## Abbreviations

AIM, activation induced marker; aAPC, artificial antigen presenting cell; CLA, cutaneous leukocyte antigen; CTV, cell trace violet; Cy5, Cy7, cyanine 5, cyanine 7; DC, dendritic cell; DEJ, dermo-epidermal junction; ECD, energy coupled dye; FRT, female reproductive tract; HPV, human papillomavirus; ICP, infected cell protein; ICS, intracellular cytokine secretion; IMGT, international immunogenetics information system; iNKT, invariant natural killer T; IVTT, *in vitro* transcription translation; EBV-LCL, Epstein-Barr virus transformed lymphocyte continuous line; LCM, laser capture microdissection; MAIT, mucosa-associated invariant T; OLP, overlapping peptides; rIL-2, recombinant IL-2; RNAseq, RNA sequencing; TCL, T cell line; TCM, T cell medium; TCRα, TCRβ, T cell receptor alpha, T cell receptor beta polypeptides; TNFRSF9, tumor necrosis factor receptor superfamily member 9; *TRA, TRB*, T cell receptor alpha, T cell receptor beta genes; T_RM,_ tissue resident memory T cell; UL, unique long region of HSV genome; VP, virion protein; *vhs*, virion host shutoff gene, protein encoded by HSV gene *UL41.*
