# Supplementary figures and images for: HSV-2-Specific Human Female Reproductive Tract Tissue Resident Memory T Cells Recognize Diverse HSV Antigens
Source: Front Immunol. 2022 Mar 31;13:867962. doi: 10.3389/fimmu.2022.867962 (PMC9009524; doi:10.3389/fimmu.2022.867962)

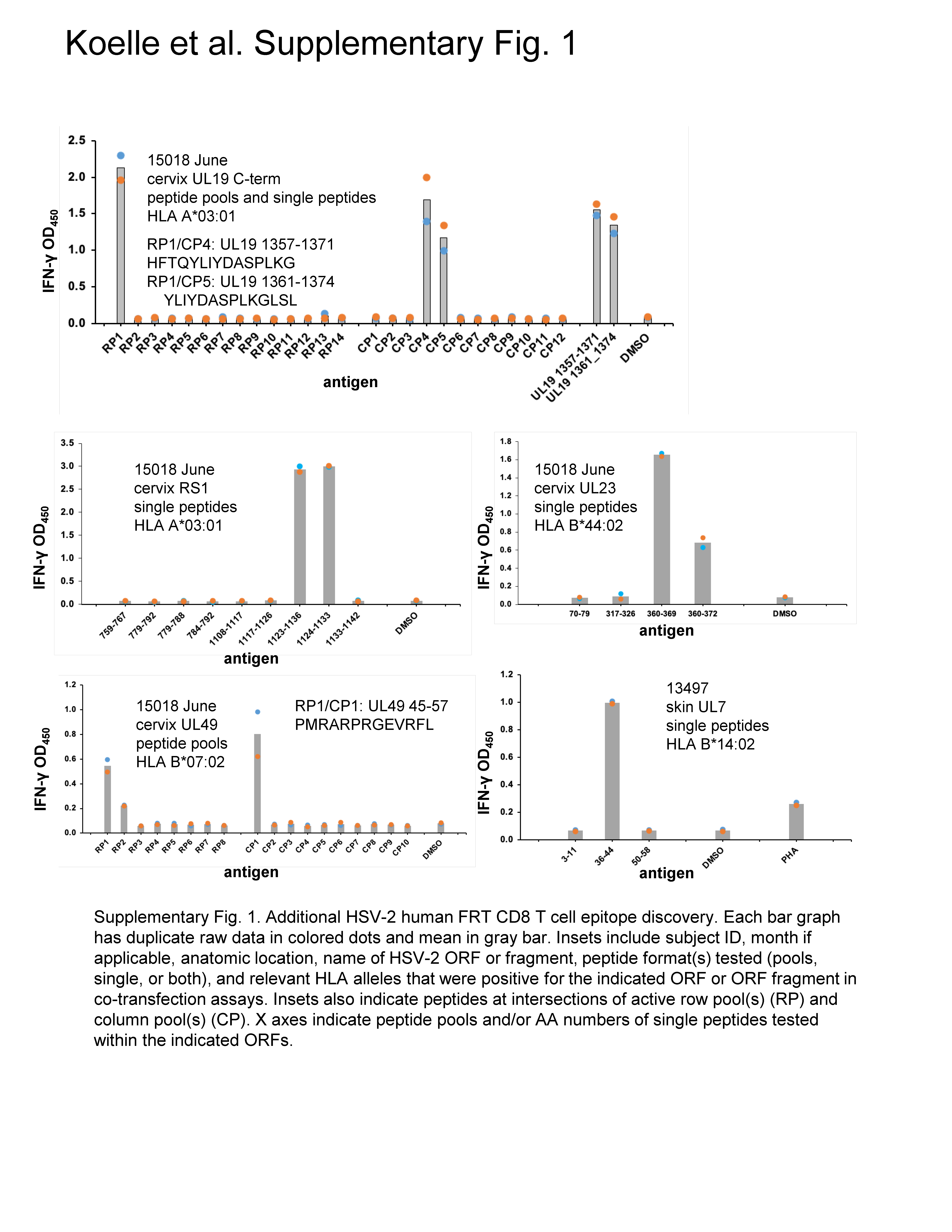

Supplement: Supplementary file 1 [file Image_1.tiff]

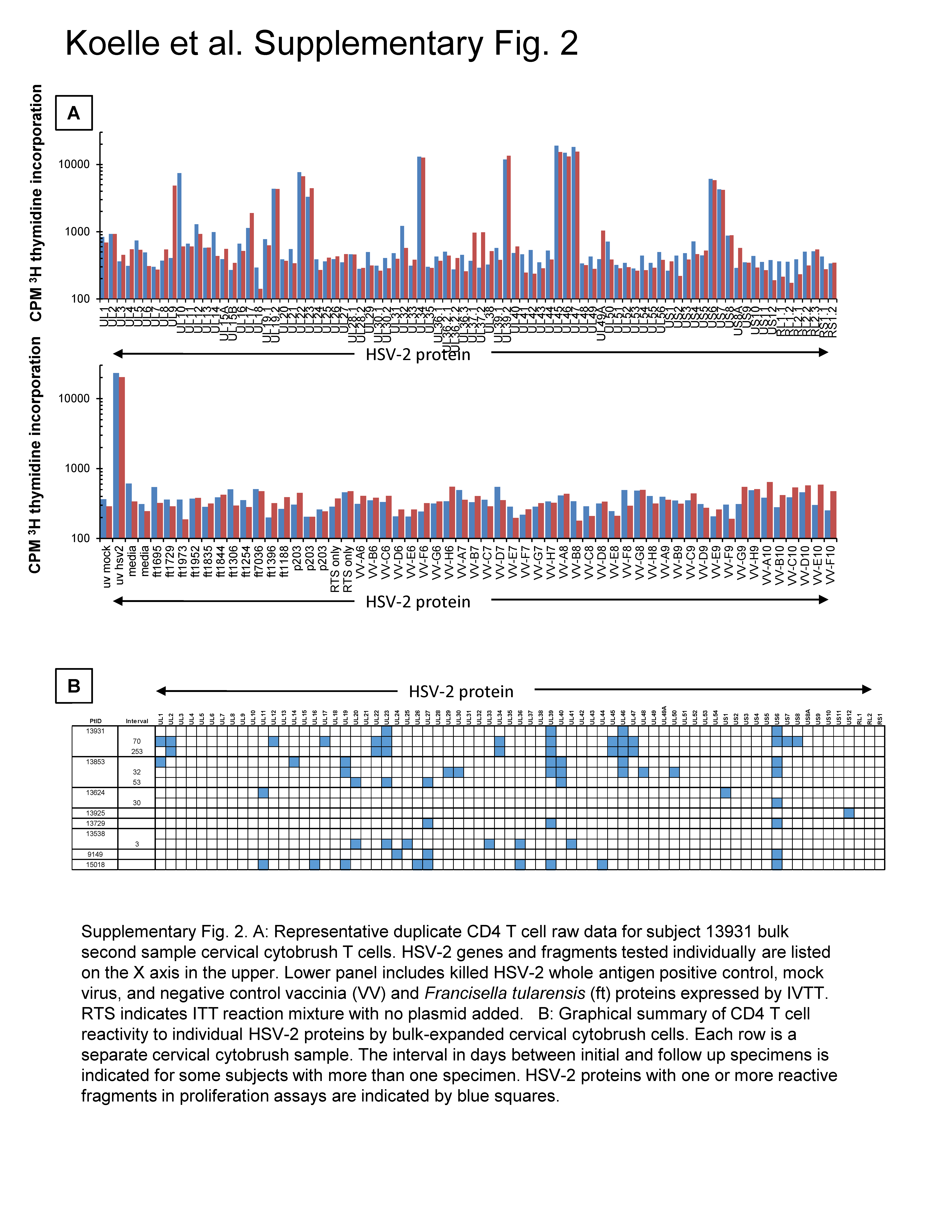

Supplement: Supplementary file 2 [file Image_2.tiff]

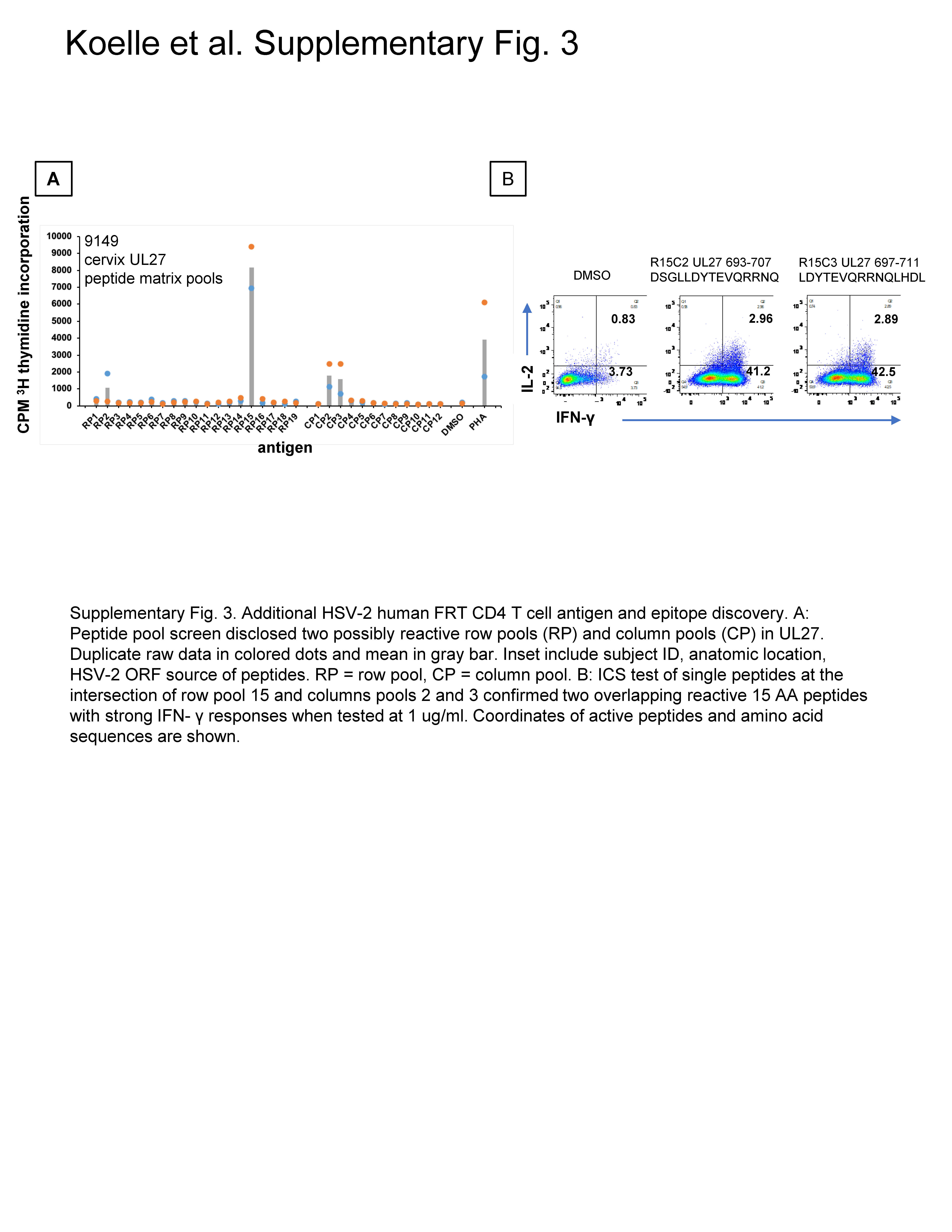

Supplement: Supplementary file 3 [file Image_3.tiff]

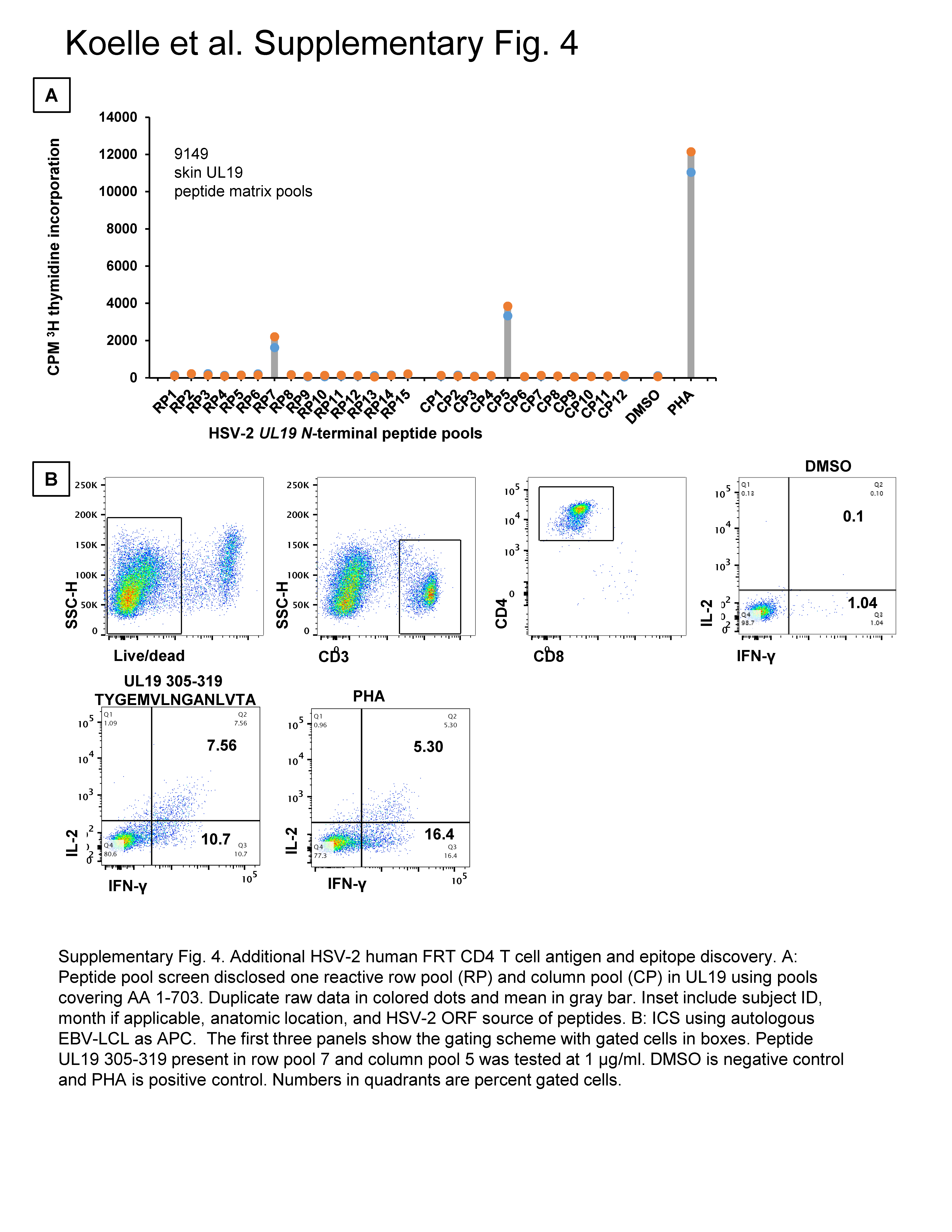

Supplement: Supplementary file 4 [file Image_4.tiff]
