# Supplementary material for: HSV-2-Specific Human Female Reproductive Tract Tissue Resident Memory T Cells Recognize Diverse HSV Antigens
Source: Front Immunol. 2022 Mar 31;13:867962. doi: 10.3389/fimmu.2022.867962 (PMC9009524; doi:10.3389/fimmu.2022.867962)
Supplement: Supplementary file 5 [file Table_1.docx]

**Supplementary Tables**

Supplementary Table 1. HLA types^1^ of biopsy participants.

|  | HLA locus | | | | | | |
| --- | --- | --- | --- | --- | --- | --- | --- |
| PtID^2^ | A | B | C | DRB1 | DRB3,4,5 | DQA1 | DQB1 |
| 5491 | 26*01 | *07:02 | *07 | *03:01 | B3 positive |  | *02:01 |
|  | *01:01 | *08:01 | *07 | *15:01 | B5 positive |  | *06:02 |
|  | | | | | | | |
| 9149 | *01:01 | *08:01 | *07:01 | *03:01 | 3*01:01 | *05:01 | *02:01 |
|  | *68:01 | *44:02 | *07:04 | *11:01 | 3*02:02 | *05:05 | *03:01 |
|  | | | | | | | |
| 13497 | *01:01 | *14:02 | *06 | *01:02 | 4*01:01 | *01:01 | *03:05 |
|  | *03:02 | *57:01 | *08 | *04:03 |  | *03:01 | *01:01 |
|  | | | | | | | |
| 14887 | not done | | | | | | |
|  | | | | | | | |
| 14655 | *02:01 | *07:02 |  | *01 | B3 positive |  | *05 |
|  | *03:01 | *40:01 |  | *13 |  |  | *06 |
|  | | | | | | | |
| 15018 | *03:01 | *07:02 |  | *04:01 | 4*01:03 | *01:02 | *03:01 |
|  | *32:01 | *44:02 |  | *15:01 | 5*01:01 | *03:03 | *06:02 |
|  | | | | | | | |
| 15052 | *02:01 | *15:10 |  |  |  |  |  |
|  |  | *35:01 |  |  |  |  |  |

^1^ Typing reported at the level of definition performed, which varied between participants. Rows do not represent haplotypes. Blank cells indicate tests that were not done.

^2^ Participant ID number.

Supplementary Table 2. Participants and specimens used for cervical cytobrush-based CD4 T cell assays.

| PtID | cytobrush date(s) | age | HSV-1^1^ | Duration of genital herpes at time of cytobrush, years | genital herpes recurrences/year^2^ | genital HSV shedding rate^3^ | day(s) since most  recent clinical herpes recurrence(s) ^4^ |
| --- | --- | --- | --- | --- | --- | --- | --- |
| 9149 | 8/31/16 | 54 | negative | 31 | 2 | 8.3 | unknown |
|  |  |  |  |  |  |  |  |
| 13538 | 10/02/12 | 50 | negative | 34 | 8 | 0.0 | 15 |
| 13538 | 10/05/12 |  |  | 34 |  |  | 18 |
|  |  |  |  |  |  |  |  |
| 13624 | 7/20/10 | 29 | negative | 5 | 12 | unknown | 10 |
| 13624 | 8/18/10 |  |  |  |  |  | 40 |
|  |  |  |  |  |  |  |  |
| 13729 | 05/31/12 | 29 | negative | 3 | 8 | unknown | unknown |
|  |  |  |  |  |  |  |  |
| 13853 | 7/19/10 | 21 | negative | 1 | 2 | 0.0 | unknown |
| 13853 | 8/20/10 |  |  | 1 |  |  | unknown |
| 13853 | 9/10/10 |  |  | 1 |  |  | unknown |
|  |  |  |  |  |  |  |  |
| 13925 | 06/06/13 | 23 | negative | 3 | 1 | 40.0 | 8 |
|  |  |  |  |  |  |  |  |
| 13931 | 7/12/10 | 32 | negative | 3 | 2 | unknown | unknown |
| 13931 | 9/20/10 |  |  | 3 |  |  | unknown |
| 13931 | 3/22/11 |  |  | 3 |  |  | unknown |
|  |  |  |  |  |  |  |  |
| 15018 | 6/27/16 | 37 | negative | 1 | 2 | 57.7 | 10 |
| 15018 | 9/28/16 |  |  | 1 |  |  | 11 |
